# Migration of nurses and doctors: pull factors to work in Saudi Arabia

**DOI:** 10.1186/s12960-023-00809-5

**Published:** 2023-03-20

**Authors:** Husam Almansour, Ameera Aldossary, Sandra Holmes, Thamer Alderaan

**Affiliations:** 1 Health Management Department, College of Public Health & Health Informatics, University of Ha’il, Ha’il, Saudi Arabia; 2 Nursing Saudization, Practice, Education & Research, King Fahad Specialist Hospital, Dammam, Saudi Arabia; 3 Human Resources Department, Dammam Health Network, Dammam, Saudi Arabia

**Keywords:** Health service management, Healthcare worker mobility, Healthcare worker migration, Job satisfaction, Multicultural issues, Recruitment, Retention

## Abstract

**Background:**

Although Saudi Arabia is a common destination to which nurses and doctors migrate, few studies have explored the pull factors attracting them to work in the Middle East and Saudi Arabia. This qualitative study explores the pull factors drawing nurses and doctors to work in Saudi Arabian hospitals.

**Methods:**

The study utilized a qualitative approach with focus groups. The participants included 83 doctors and nurses at two government hospitals.

**Results:**

Five themes (rewards, job entry requirements, religion, influence of family and friends, and changing work environments) were identified based on the 10 focus group sessions.

**Conclusion:**

Moving forward, health managers should proactively plan the state of healthcare as the need for migrant healthcare workers changes.

## Background

People have always migrated. Individuals and groups have moved from one place to another to survive. Migration has had to occur to meet the needs for safety, food, water, and opportunities. In the last century, healthcare migration has become more common. As the need for healthcare providers has grown, countries have become more engaged in recruiting migrant healthcare workers. About 50 years ago, the World Health Organization (WHO) called for an investigation into the causes of healthcare migration and a plan to resolve the problems caused by it [1]. This investigation found that the main reasons for healthcare migration were the social and economic problems with healthcare systems in many countries, such as poor planning of the health workforce, low job satisfaction, political and economic instability, discrimination, and corruption [1]. These central, intertwined push factors from donor countries and pull factors from recipient countries appear to mirror each other and have driven healthcare workers’ migration accordingly.

Migrant healthcare workers, who come from many different places, contribute significantly to the health system’s workforce where shortages exist. Looking at global physician mobility, the main donor countries of physicians to the world include India, Pakistan, China, Germany, Romania, the Russian Federation, the United Kingdom, Poland, Iran, Italy, and the Philippines [2]. The Philippines is the largest donor of nurses (30%), followed by India (6%) and Nigeria (5%) [3]. Other major exporters of healthcare workers are Mexico, Malaysia, Colombia, Egypt, and Pakistan. At the same time, the importers are mainly high-income countries, such as New Zealand, the USA, the UK, Canada, and Australia [4, 5]. The WHO adopted a Global Code of Practice on the International Recruitment of Health Personnel in 2010 to “strengthen the understanding and ethical management of international health personnel recruitment through improved data, information, and international cooperation” [6]. The WHO code recommends that countries strive for self-sufficiency in their healthcare workers by training and retaining enough healthcare workers to meet demand. The global code notes that if recruitment is appropriately managed, the international migration of health personnel can contribute to developing and strengthening health systems without draining resources [6].

A range of studies has investigated healthcare workers’ migration. In a study conducted in Germany, about 30% of physicians who participated wished to emigrate; the participants’ favorite destination countries were Switzerland, the Scandinavian countries, Australia, and New Zealand [7]. In Ireland, about 35% of national junior hospital doctors planned to leave the country but return later, 17% planned to leave and not return, and 3% planned to quit the medical profession [8]. The worldwide shortage of nurses, expected to total around nine million people by 2030, is of great concern [9]. The issue of nurses emigrating to other countries creates a drain on existing resources, especially in low-income countries with greater needs for healthcare workers. Nurses seeking to leave due to unsatisfactory salaries or benefits, a lack of professional development, and better work opportunities in other countries have been reported in recent studies [10, 11].

Factors that force individuals from one place and attract them to others are called “push” and “pull” factors, respectively [12]. Push factors are usually associated with donor countries; pull factors are identified with receiving countries. Both are commonly identified and reported in the literature on migration. The forces behind the factors are typically identified as political, social, economic, legal, historical, cultural, and educational [1]. Individuals assess push and pull factors as they attempt to find a safer place to live, a better quality of life and leisure, employment, and professional development opportunities for themselves and their families. The increased opportunities to gain credentials in another country or the ease of doing so, due to changes in recruitment and governmental processes support emigration. These opportunities are identified as driving factors for healthcare workers, especially those in lower-income countries [13].

Saudi Arabia is considered one of the primary recipient countries of healthcare workers’ migration, importing staff from multiple countries [14]. In Saudi Arabia, healthcare services are administered primarily through the Ministry of Health (MOH) and other governmental entities, i.e., military hospitals and private facilities. Per the latest MOH Statistical Yearbook [15], there were 287 MOH hospitals, 51 other governmental facilities, and 159 private sector facilities. These hospitals reported 45 330, 14 005, and 17 889 beds, respectively. Additionally, 2257 primary healthcare centers (PHCs) were reported [15]. A total of 122 356 doctors and 201 489 nurses and midwives were responsible for providing care in these facilities. Migrant healthcare workers in the Ministry of Health account for 50% of the doctors and 37% of the nursing staff [15]. While there has been an increase in the number of Saudi nationals serving in the health sectors over the past two decades, there is still a reliance on migrant healthcare workers in some specialties, which is common even among some developed countries that recruit from other countries. Doctors migrate from different countries that enjoy peaceful and cooperative relationships with Saudi Arabia, but mainly come from Egypt, Jordan, Sudan, India, and Pakistan. Nurses mainly come from the Philippines and India.

Generally, healthcare workers are actively recruited to work in Saudi Arabia via government listings and recruitment agencies. Interested individuals can identify positions in Saudi Arabia through career or job postings on media platforms, professional associations, or career listing boards, such as LinkedIn, MonsterJobs, etc. Once a candidate for a position has been interviewed and vetted through a review of credentials and status (legal, health, etc.) by the Saudi Embassy and hospital human resources department, they are invited to join a facility. Once they are in the country, professional documents and fees are submitted to the professional credentialing agency, and the appropriate license and designation are conferred. There are some discrepancies in which healthcare migrant workers obtain family or single contracts. These depend on several factors, such as the type of hospital (i.e., government hospitals, specialized hospitals, private hospitals), job positions, and allocated budgets at the time of recruitment.

The situation in Saudi Arabia used to be different from those in other recipient countries before the establishment of the Premium Residency Program [16], as historically, migrant healthcare workers worked on a temporary basis with minimal opportunities to obtain permanent residency or citizenship. Although some attractive measures are helping the system, there needs to be further research regarding the pull factors that influence the migration of healthcare workers to work in Saudi Arabia. Therefore, this study aims to identify the current pull factors drawing doctors and nurses to work in Saudi Arabian hospitals.

## Methods

### Design, sample, and setting

A descriptive qualitative approach utilizing focus group discussions (FGs) conducted in government hospitals was taken to explore the pull factors influencing the decision to immigrate to Saudi Arabia. This approach helps explore phenomena and problems relating to people’s experiences and perceptions [17]. The inclusion criteria consisted of being a registered migrant nurse or doctor with at least one year of experience working in a Saudi hospital. Unlicensed and unemployed nurses and doctors and those not affiliated with a hospital were excluded. The recruitment process for the FGs involved sending an invitation email to a convenient sample of migrant nurses and doctors to participate in the study. Participants who were willing to take part in the FGs were invited based on convenient times to meeting rooms within hospitals. Ten focus group discussions (FGs) were convened with 83 nurses and doctors from different specialties at two government hospitals in Saudi Arabia between March and July 2020. The groups ranged in size from six to ten participants per session based on their practice backgrounds to ensure homogeneity and to capitalize on their shared experiences [18]. The participants were purposively selected for the focus groups to confirm that a sufficient number of both doctors and nurses participated in the study. The final number of participants was determined by saturation. The FGs were stopped when the researchers agreed that data categories were established and new data fit into already developed categories.

### Data collection

The FGs were organized to collect information about pull factors that influenced the decisions to immigrate of nurses and doctors. An open-ended question regarding the study’s aim asked nurses and doctors about pull factors to work in Saudi Arabia. Follow‐up questions such as “What do you mean?” or “Can you clarify, please?” were asked to solicit additional information and encourage additional discussion. The focus group sessions were conducted by one of the researchers (TA) in English, as it is the primary language in healthcare settings in Saudi Arabia. All participants were fluent in English regardless of their country of origin or mother languages. The interviewer who acted as the moderator of the FGs ensured the involvement of all participants by asking about their perceptions of the research objectives. This was to ensure that all voices were heard and to avoid the domination of FGs by certain individuals, which is considered a common challenge in the dynamics of focus group methods. In this study, however, the participants shared many common features. They were all migrant workers and their pull factors were consistent in several aspects, which helped the interviewer manage the FGs successfully.

Participation was voluntary, and all the participants were assured confidentiality and anonymity. The sessions were voice-recorded to obtain full details from the FGs. Each session lasted about 1 h. Informed consent was gained from the participants before each FG session. The information is reported anonymously, as pseudonyms and codes are used. Ethical approval was gained from the institutional review board of a university in Saudi Arabia (H-2020-046).

### Data analysis

The audio-recorded data were transcribed to allow computerized storage and organization. The thematic analysis used followed the framework of Braun and Clarke [19]. This framework, which includes noticing and looking for patterns of meaning and issues of potential interest in the data, guided our review. Creating initial codes, searching for themes, reviewing themes, and defining and naming themes followed this phase [19]. The coded data were arranged and grouped based on similarities, and each category of topics was given a name. The text was sorted and the coding supported using MAXQDA 2020 software.

## Results

### Characteristics of participants

There were 51 females out of the total 83 participants in the focus groups. Forty-nine of the participants were nurses; 34 were doctors. Most participants had more than 10 years of experience in their field. Table 1 presents the distribution of the demographic characteristics of the focus group participants.

**Table 1:** Distribution of the demographic characteristics of the focus groups participants

Demographic data	Frequency
Gender	
Female	51
Male	32
Country of origin	
Filipino	21
Indian	19
Egyptian	14
Jordanian	8
Sudanese	7
Pakistani	6
Malaysian	4
South African	4
Religion	
Muslim	64
Non-Muslim	19
Profession	
Nurses	49
Doctors	34
Years of living and working in Saudi Arabia	
< 5	10
5–10	29
> 10	44

### Pull factors of working in Saudi Arabia

Pull factors to work in Saudi Arabia can be represented by five themes that developed from the findings of the focus groups. These themes included rewards, job entry requirements, religion, the influence of family and friends, and changing work environment.

#### Theme 1: Rewards

The competitive reward package in Saudi Arabia was the primary pull factor for migrant nurses and doctors. All participants indicated that Saudi salaries were higher than those in their home countries. The salary in Saudi Arabia was as much as about four times higher than salaries back home. The migrant nurses and doctors reported spending money on buying houses in their home countries, supporting their children, and sending them to school or college. Additionally, migrant healthcare workers enjoy approximately 60 days paid vacation and holidays, free annual flights to visit their home countries, housing or a housing allowance, a travel allowance, and sometimes, family contracts that cover children under 18 years of age. Four participants illustrated these points by discussing the importance of the rewards to their family finances back home.

*The offered package was one of the most influential reasons to come to Saudi Arabia. The salary is more than four times my salary in Egypt, plus I get free annual flight tickets and an accommodation package.* (Egyptian doctor, Hospital B.)

*Salaries are much greater than those available to me in my home country and many other countries, allowing greater opportunity to save.* (Indian doctor, Hospital A.)

*I can earn a higher salary in Saudi Arabia. My kids are growing up, and I want them to have a good education and better quality of life. So, I decided to come to Saudi Arabia, where I receive a higher salary.* (South African nurse, Hospital A.)

*My classmates are working in America, Canada, and Ireland. They receive no paid vacation or flight tickets to the Philippines, which are very expensive. They must pay for their own flights and are not paid during time off.* (Filipino nurse, Hospital B.)

#### Theme 2: Job entry requirements

Some participants reported that one of the pull factors of working in Saudi Arabia was the belief in the simplicity of landing a job there compared to other countries. Those participants listed some of the problematic requirements needed to land a job in some Western countries, such as taking board exams and language tests, having extensive experience, and providing specific documentation. On the other hand, some participants noted that the requirement to work in Saudi Arabia was more straightforward than in their home countries, especially when seeking employment in prestigious settings, such as government hospitals. Some participants decided to work in Saudi while working on their immigrant visas and jobs in other countries. Others intend to return to their home countries after a certain length of time in Saudi Arabia. However, participants believed Saudi Arabia was a better option since it did not require extensive experience or any difficult exams when they applied for their jobs.

*The requirements for working in Saudi Arabia are not complicated, which is why I chose to work there.* (Sudanese doctor, Hospital A.)

*In Saudi Arabia, work requirements are less difficult to meet than those of many Western countries, which require a long process that includes language tests, difficult certification exams, and other documentation. Saudi Arabia hires young nurses with less experience. By not requiring years of experience, they offer more opportunities and accept more people.* (Indian nurse, Hospital B.)

*Gaining employment in the Filipino government hospitals is difficult, as positions are very limited. I applied to immigrate to the United States, but the process takes a long time. After about five years waiting for an immigrant visa to the United States, I ended up working in Saudi Arabia.* (Filipino nurse, Hospital A.)

#### Theme 3: Religion

About 80% of Muslim participants said that religion was one of the pull factors for working in Saudi Arabia. Mecca and Medina (the Kaaba and the Noble Prophet’s Mosque) are in Saudi Arabia. Muslim participants noted that working in Saudi Arabia allowed them to earn money for their families and visit these locations periodically. Participants stated that it could be difficult and costly to travel to Saudi Arabia to attend important pilgrimages (Hajj and Umrah) when living overseas. As residents of Saudi Arabia, migrant Muslim nurses can practice their religion, obtain visas for their families to visit, and perform religious duties.

*I chose Saudi Arabia over other Gulf countries because in Saudi Arabia, I can periodically visit Mecca and Medina.* (Egyptian doctor, Hospital A.)

*In Malaysia, one must wait for a turn to join the Hajj, so I would need to get on a list. Now, I work and can perform Umrah. I have performed Umrah countless times—nearly three or four times per year.* (Malaysian nurse, Hospital B).

#### Theme 4: Recommendations of family and friends

Some participants discussed the influence of family and friends as a pull factor to work in Saudi Arabia. They emphasized the influence of their friends or family members who had worked in Saudi Arabia and recommended working there. For example, two participants explained this situation:

*I have many friends and relatives in the Kingdom of Saudi Arabia who advised me to go work in Saudi Arabia.* (Sudanese doctor, Hospital B.)

*I was most influenced by my best friend, who has been working in Saudi Arabia since 2002. I trust her. She’s been my best friend since high school.* (Filipino nurse, Hospital A.)

#### Theme 5: Changing the work environment

The pull factors for working in Saudi Arabia included increasing knowledge and skills and gaining experience in a different work environment, which the nurses and doctors believed they needed to move on in their careers, as well as exposure to allow them to grow. In addition, some participants indicated that they came to Saudi Arabia to be exposed to a different culture. They had worked at their previous hospitals for many years and wanted to change. Three participants summarized these views:

*We develop all the necessary skills. Most of the nurses in Saudi Arabia gain experience and then seek better opportunities in Australia or New Zealand.* (Filipino nurse, Hospital A.)

*One reason I came to Saudi Arabia was to change my work environment. I had spent 10 years at the same hospital in Jordan, so I felt the need for change.* (Jordanian doctor, Hospital A.)

*Having been at the same hospital for 17 years, I was really in a rut. I also wanted to see how the other side worked and lived. My decision was probably based half on money and half on the opportunity to work in a different environment.* (Filipino African nurse, Hospital B.)

Changes in the working environment were pull factors for working in Saudi Arabia. The country has newly developed facilities and technology along with a better health infrastructure compared to home countries with less developed economies, such as the Philippines, India, Pakistan, Egypt, and Sudan.

*The work environment is good and encourages higher medical standards because it includes advanced facilities and technologies. (*Pakistani doctor, Hospital A.)

*Working conditions in the health facility are comfortable.* (Filipino nurse, Hospital B.)

## Discussion

The themes identified by the participants in this study identified pull factors for nurses and doctors interested in working in Saudi Arabia. Some of these factors were consistent with those seen in other countries. For example, one of the primary reasons for all participants to move to Saudi Arabia was greater rewards, including salaries and other financial benefits. The increased income allows them to save more and send more money back to their home countries than they would otherwise have to spend there. This is in line with the sentinel study by Mejia [1], which reported greater rewards as a leading cause of nurses’ and physicians’ migration.

The simplicity of gaining employment was another attraction to Saudi Arabia. This study revealed fewer challenges to getting employed in the Kingdom compared to other developed countries. In a recent investigation of Syrian doctors who work in Germany conducted by Loss et al. [20], doctors reported that the licensure procedure was complicated, lengthy, ever-changing, and non-transparent. In a study of Indian and Filipino nurses who work in the UK, participants considered Saudi Arabia a steppingstone to migrate to their favored destination, as they can pass job entry requirements, gain experience, and work on other requirements to move to other countries [21].

One of the unique pull factors attracting nurses and doctors to move to Saudi Arabia is better access to participation in religious practices. The Hajj pilgrimage, one of the pillars of Islam, is performed at a strictly designated time of the year, whereas Umrah, another practice, can be performed at any time. Some migrant Muslim nurses and doctors seek employment in Saudi Arabia to fulfill their religious beliefs instead of being held on a waiting list to perform religious practices. This opportunity is an advantage, as millions of Muslims worldwide are waiting for their turn in the areas where the sacred rites and rituals take place.

Moving to a foreign location requires giving up the security of having family, friends, and an environment that one has known and been comfortable with. This study revealed that some migrant nurses and doctors select Saudi Arabia based on the recommendations of family and friends. This in in line with Alonso and Maben [21] and Nair and Webster [5], who identified social factors that influence some health workers to decide to immigrate. The influence of family and friends is significant and could be rooted in human nature, not only in worker migration, but also in selecting a profession to begin with and other daily activities [22]. Family and friends who have experience working in Saudi Arabia generate some of the best recruitment of new workers, as they can share information about living and working here. Additionally, they provide social support and ease the transitions of new workers once they arrive.

Migrant nurses and doctors move to Saudi Arabia to gain additional competencies in a healthier, developing, progressive environment. This is consistent with Nair and Webster [5], who described the infrastructures and medical technologies available in the healthcare systems in emerging market economy countries as often less advanced, pushing healthcare workers to migrate to areas that are more developed. Saudi has a modern, technologically advanced healthcare system. Most major facilities belong to the Joint Commission International or Saudi Central Board for Accreditation of Healthcare Institutions and hold other specialty accreditations.

There is a major transformation occurring in Saudi Arabia that affects all sectors. Reliance on Saudi nationals to assume greater responsibility for leading and supporting work in the Kingdom is paramount. Specifically in the healthcare arena, there are plans to ensure that the system is based on supporting the health of the individual and society, is financially sustainable, and assures access to care with optimal coverage, high quality, and safety in a satisfying manner [23]. The Vision of Saudi Arabia for 2030 requires an increase in Saudi medical and nursing graduates prepared to assume healthcare positions. The numbers and types of migrant nurses and doctors will change over time. Newly qualified resident doctors in Saudi Arabia often seek training in specialized areas, and some of these opportunities are limited [24]. While the reliance on migrant trained nurses and doctors will decrease, there will still be pull factors that attract individuals to Saudi Arabia. Buchan et al. [22] suggested that sustainability must consider migrant healthcare workers’ input and be clear about any upcoming adjustment of this level. This does not mean the country should rely only on locally trained nurses and doctors. Buchan et al. [22] suggested that globally, current healthcare workers should be used more effectively and requires continual development, retraining, and more focus on primary care-led services to enable improved access for the population. Through effective recruitment and retention procedures, enhanced technological solutions, and artificial intelligence, more staff can be deployed in rural and underserved areas [25].

The Global Strategy on Human Resources for Health has stated that the ability to successfully match the supply and skills of the healthcare workforce to the needs of the population is a priority in the twenty-first century [25]. Greater control of the dynamics of labor markets is needed to ensure that high levels of active, long-term international recruitment have no damaging effect on less well-resourced countries by depriving them of scarce skilled workers.

The scope of this study was to explore pull factors to work in Saudi Arabia. A previous study explored job dissatisfaction among expatriate nurses compared to nurses in their home countries and revealed factors such as language barriers and separation from family and friends, which are common among migrant healthcare workers [26]. Healthcare workers emigrating to Saudi Arabia are not without challenges. This is especially true for young, non-Muslim, and non-Arabic speaking workers. There is a general period in which one must gain cultural awareness and competency if never exposed to living and working in the Middle East, just as there would be when transitioning to any other country. The options and opportunities within Saudi Arabia are expected to remain viable, even as Saudi works to prepare its nationals to fill positions and reduce its reliance on migrant healthcare workers.

## Strengths and limitations of the study

This study has aimed to improve our knowledge regarding the migration of nurses and doctors by exploring pull factors to work in Saudi Arabia, as there are limited studies in this context. This qualitative study followed the framework used by O’Brien et al. [27] to report qualitative research. The main limitation of the qualitative approach is that the results cannot be generalized. This limitation is typical in qualitative study designs, as they involve small sample sizes. Additionally, this study did not focus on the exploration of challenges during migration alongside attractors to work in Saudi Arabia. While the themes identified in this study align with those in other areas, there are specific religious factors that are not associated with international migration in general.

## Conclusions

This study explored the pull factors of nurses and doctors seeking employment in Saudi Arabia. It revealed that these factors include rewards, job entry requirements, religion, recommendations of family and friends, and changing work environments. Working in Saudi Arabia can be a positive experience with long-term financial and career benefits. Additionally, those interested in taking advantage of being in the country can participate in religious practices that might not otherwise be easily accessed. Given the transformation of the healthcare system, the number of Saudi doctors and nurses assuming more positions will reduce the need for immigrant workers, and some pull factors, especially salary, and benefits, may grow less attractive. Moving forward, healthcare managers and potential healthcare workers should proactively plan for the future state of healthcare as the need for migrant healthcare workers changes. As mentioned in the Global Code of Practice on the International Recruitment of Health Personnel [6], the health of all people is essential to achieve peace and security and is dependent upon the fullest cooperation of individuals and states. Moreover, the positive impacts of migration and mobility on individuals, such as an improved financial situation and acquisition of experience in a new working environment, should be considered when developing international strategies and policies governing health worker migration. Another factor to consider is the positive impacts of migration on source countries, such as the transferred remittances that improve the economies of immigrants’ home countries.

## Data Availability

Due to privacy and ethical concerns, neither the data nor the source of the data can be made available.
